# Leveraging near-peer teaching: the impact of cognitive and social congruence on naloxone training

**DOI:** 10.1080/10872981.2026.2676337

**Published:** 2026-05-19

**Authors:** Jachua Polcyn, Robert VanBrackle, Natalie Zink, Katherine Covington, Vahé Heboyan, Nicole Rockich-Winston

**Affiliations:** a Medical College of Georgia at Augusta University, Augusta, GA, USA; b Department of Emergency Medicine, University of Chicago, Chicago, IL, USA; c Institute of Public and Preventative Health, Augusta University, Augusta, GA, USA; d Department of Health Management, Economics, & Policy; Institute of Public and Preventative Health, Augusta University, Augusta, GA, USA; e Department of Pharmacology and Toxicology, Medical College of Georgia at Augusta University, Augusta, GA, USA

**Keywords:** Naloxone, opioids, near-peer teaching, cognitive congruence, social congruence

## Abstract

**Background:**

Equipping future clinicians to recognize and reverse opioid overdoses requires experiential training and access to naloxone. Near-peer instruction may leverage cognitive and social congruence to enhance learning compared with traditional faculty-led models. This study evaluated whether near-peer teaching yields superior knowledge gains and attitudes in a naloxone training workshop.

**Methods:**

Preclinical medical students completed a 60-minute session comprising a high-fidelity inpatient opioid overdose simulation and hands-on naloxone administration practice. Immediately pre- and post-workshop, participants completed the Opioid Overdose Knowledge Scale (OOKS) and Opioid Overdose Attitudes Scale (OOAS). We compared near-peer–led versus faculty-led sessions using descriptive statistics and independent t-tests. Using an explanatory sequential design, 10 students completed semi-structured interviews exploring preferences for near-peer teaching. Interviews were transcribed and analyzed using grounded theory, applying the concepts of cognitive and social congruence as an analytical lens.

**Results:**

From 2019 to 2022, 537 students attended the workshop, and 288 students provided complete survey data. Near-peer facilitation was associated with higher OOKS scores (84.5% ± 0.09 vs 80.2% ± 0.10 correct; *p* < 0.001) and more favorable OOAS totals (105.5 ± 8.4 vs 102.4 ± 13.7; *p* = 0.02) compared with faculty-led sessions. Interviewees highlighted a shared knowledge framework, familiar language/explanations, and understanding of learning challenges. Additionally, interviewees noted informal communication, reduced anxiety, and interest in near-peer students’ experiences.

**Conclusion:**

A brief, near-peer–led overdose simulation and naloxone skills workshop was associated with an improvement in medical students’ knowledge and attitudes compared to faculty-led delivery. Integrating trained near-peer educators into preclinical curricula may accelerate acquisition of overdose recognition and response skills while promoting clinical readiness. Programs seeking scalable strategies to expand skill-based, practical competencies should consider near-peer models that benefit from students’ diverse prior experiences.

## Introduction

Opioid-related overdose deaths have been steadily increasing, with recent data indicating over 79,000 deaths in 2023 [1]. Although naloxone was approved as a reversal agent for opioid overdoses in the 1970s, its use for overdose treatment was restricted to emergency medical personnel in emergency departments and paramedics in the field [2]. Since then, policy makers and harm reduction advocates have focused on making naloxone more accessible, establishing distribution programs at syringe exchanges, pharmacies, and through emergency medical services (EMS) personnel [3].

Training medical students on how to recognize the signs and symptoms of an opioid overdose and effectively treat individuals experiencing an opioid overdose offers a salient way to increase the number of healthcare personnel available to them [4–6]. Additionally, medical students with previous EMS training are a valuable resource for training their peers [7]. While recent literature proposes that peer-assisted teaching in preclinical medical education may result in similar outcomes compared to faculty-led or other professionally based teaching [8], near-peer instruction capitalizes on shared educational frameworks and relational familiarity, more commonly known as cognitive and social congruence [9]. Cognitive congruence refers to shared knowledge frameworks among near-peer teachers and learners, while social congruence emphasized the similar social roles among such groups. Additionally, significant improvements have been demonstrated in peer-assisted learning during clinical medical training [8].

The Medical College of Georgia has recently redesigned our annual naloxone training from a faculty-led to a near-peer teaching workshop leveraging the EMS backgrounds of current medical students. As such, this study examined differences in knowledge acquisition and attitudinal changes between peer-led and faculty-led naloxone workshops.

## Methods

Preclinical medical students completed a 60-minute session comprising a high-fidelity inpatient opioid overdose simulation and hands-on naloxone administration practice as previously described and presented [10]. Training backgrounds for the faculty-led session included a board-certified emergency medicine physician and a pharmacist (2019), while the near-peer teaching sessions were led by a medical student with four years of paramedic experience (2021−2022). Data from 2020 were excluded due to having to convert the training to a virtual format during the COVID pandemic lockdown (March 2020).

### Workshop content and learning objectives

The workshop was designed using a structured pedagogical framework informed by experiential learning theory [11]. The session was organized into two phases: a 30-minute hands-on naloxone administration practicum using intransal and intramuscular delivery devices that also reviewed opioid pharmacology, overdose pathophysiology, and the mechanism of action of naloxone; and a 30-minute high-fidelity simulation of an inpatient opioid overdose scenario. The key learning objectives for the workshop were to (1) recognize the signs and symptoms of opioid overdoses; (2) demonstrate appropriate assessment of an unresponsive patient; (3) administer naloxone by the appropriate route; (4) describe the pharmacokinetics and duration of action of naloxone; and (5) describe the appropriate follow-up care and monitoring for patients receiving naloxone. These objectives were mapped to items the surveys described below and institutional competency-based objectives.

Both faculty-led and near-peer-led sessions followed identical content outlines and used the same equipment and naloxone administration devices. Session facilitators received a standardized facilitator guide detailing learning objectives, simulation script, key teaching points, and hands-on practice procedures. The near-peer facilitators completed a train-the-trainer session with faculty to ensure consistency in content delivery and practical skill demonstration.

### Study methodology

This mixed method study incorporated an explanatory sequential design, first collecting and analyzing quantitative results, followed by qualitative data collection and analysis. Post-tests/surveys were administered via the Qualtrics platform including the Opioid Overdose Knowledge Scale (OOKS) and the Opioid Overdose Attitudes Scale (OOAS) [12]. The OOKS instrument consists of 45 items that focus on knowledge related to naloxone administration, pharmacokinetics, risk factors, opioid overdose signs/symptoms, and opioid overdose management [12]. The OOAS survey is a 28-item instrument focusing on competencies involving opioid overdose management, concerns, and readiness to intervene during a suspected opioid overdose [12]. Due to differences in data capture between cohorts, two items were removed from analysis from two different subscales, one item from the ‘Concerns About Intervening’ and one item from the ‘Readiness to Intervene’ subscales. The decision to remove these two items was necessitated by an inadvertent omission in the 2021 and 2022 cohorts. While this modification slightly alters the original instrument structure, the remaining items within each subscale retained adequate internal consistency (Cronbach’s alpha > 0.7 for all subscales), and total scale scores maintained their validity as measures of opioid overdose attitudes.

Descriptive statistics and independent t-tests assessed for significant differences (*p* < 0.05) using SPSS v.28. Prior to conducting independent t-tests, the assumption for normality was evaluated using the Kolmogorov-Smirnov test for each outcome variable. For the OOKS total score, the near-peer group (D = 0.084, *p* = 0.15) and the faculty-led group (D = 0.1, *p* = 0.21) demonstrated non-significant deviations from normality. Similar results were obtained for the OOAS total score (near-peer group, D = 0.056, *p* = 0.62; faculty-led group, D = 0.052, *p* = 0.73), supporting the use of parametric tests.

In the subsequent qualitative analysis, 10 students were recruited for interviews to explore why students prefer near-peer teaching. J.P. and R.V. conducted semi-structured interviews that were recorded, transcribed and analyzed using grounded theory. Data collection was conducted iteratively in accordance with grounded theory principles, whereby initial findings informed theoretical sampling decisions and prompted investigation of additional conceptual themes [13]. The concepts of cognitive and social congruence were then applied to organize and inform the analysis further by J.P., R.V., and N.R-W [9]. Qualitative data analysis was completed using Dedoose, a web application for mixed method studies (SocioCultural Research Consultants, LLC, Los Angeles, California).

Ethical approval was obtained from Augusta University’s IRB (#1600134 and #2326703). Written informed consent was obtained from all participants for the quantitative survey data collection. Verbal informed consent was obtained from all participants for the qualitative interviews; verbal consent was employed for the interviews to preserve the anonymity of participants, so no documentation could be traced back to participants. Additionally, this study was conducted in accordance with the ethical principles outlined in the Declaration of Helsinki.

## Results

From 2019 to 2022, 537 students attended the workshop, and 288 students provided complete survey data (response rate = 53.6%). See Table 1 for OOKS and OOAS survey results.

**Table 1.** OOKS and OOAS results comparing faculty-led teaching versus near-peer.

OOKS	Faculty-Led Percent Correct (SD) (*n* = 106)	Near-Peer Percent Correct (SD) (*n* = 182)	*P*-Value
**Overdose Risk Sub-scale**	77.6 (0.2)	88.0 (0.2)	<0.001
**Signs of Overdose Sub-scale**	75.6 (0.1)	81.9 (0.1)	<0.001
**Life Saving Actions Sub-scale**	86.5 (0.1)	92.8 (0.1)	<0.001
**Naloxone Use Sub-scale**	80.4 (0.1)	77.9 (0.1)	<0.001
**OOKS Total**	80.2 (0.1)	84.5 (0.1)	<0.001
**OOAS**	**Average (SD)**	**Average (SD)**	
**Competence to Respond to an Overdose Sub-scale (score range: 10−50)**	38.7 (5.3)	37.7 (3.1)	0.044
**Concerns About Intervening Sub-scale (score range: 7−35**	26.7 (4.2)	28.0 (3.6)	0.006
**Readiness to Intervene Sub-scale (score range: 9−45)**	36.7 (6.3)	39.9 (3.8)	<0.001
**OOAS Total (score range: 26−130)**	102.4 (13.7)	105.5 (8.4)	0.02

*OOKS.* Medical students taught in near-peer led sessions demonstrated significantly greater knowledge of opioid overdose on three of the four OOKS subscales (Overdose Risk, Signs of Overdose, and Life-Saving Actions) than those taught in faculty-led sessions, *p* < 0.001. In addition, the total OOKS score of near-peer led sessions was significantly greater than faculty-led sessions (percent correct = 84.5% ± 0.1 versus 80.2% ± 0.1, *p* < 0.001). On the Naloxone Use subscale only, faculty-led sessions had significantly greater knowledge than near-peer led sessions (percent correct = 80.4% ± 0.1 versus 77.9% ± 0.1, *p* < 0.001).

*OOAS.* Medical students taught in near-peer led sessions had significantly more positive attitudes toward opioid overdose on two of the three OOAS subscales (Concerns About Intervening, Readiness to Intervene) than those taught in faculty led sessions, *p* = .0006 and < 0.001, respectively. In addition, the total OOAS score of near-peer led sessions was significantly greater than faculty-led sessions (105.5 ± 8.4 versus 102.4 ± 13.7, *p* = 0.02, [scale points can range from 26-130]). On the Competence to Respond to an Overdose subscale only, faculty-led sessions had more positive attitudes than near-peer led sessions (38.7 ± 5.3 versus 37.7 ± 3.1, *p* = 0.044).

*Qualitative Data.* Tables 2 and 3 illustrate example quotes for each of the main themes for cognitive and social congruence concepts. Students highlighted a shared knowledge framework, familiar language/explanations, and an understanding of learning challenges, aligning with cognitive congruence concepts. For example, one student explained, ‘They incorporated some strategies for engagement by being able to call people by name, talk more conversationally rather than having it more of a lecture’ (student #7). Additionally, students emphasized the informal communication style, reduced anxiety, and an interest in near-peer students’ experiences, supporting social congruency. As another student described, ‘[I] definitely felt more comfortable asking them questions compared to faculty’ (student #9).

**Table 2.** Cognitive congruence themes.

Theme	Representative Quotes	Student #
**Calibrated to learner level and familiar language**	“They made it very succinct…and kept it relevant to our level of expertise that we have right now.”	2
	“They incorporated some strategies for engagement by being able to call on people by name, talk more conversationally rather than having it more of a lecture.”	7
	“I think it helped me because it helped me relate to the presenter…[it] felt like it was my friends teaching me.”	8
**Understanding learner challenges/anticipation**	“I think [near-peer teaching works] because they know what module that we’re at. They’re able to ask things that relate to our learning.”	5
**Clear, specific explanations of key content**	“They did a good job of going into detail about the different routes of administration and why we would prefer to use on over the other.”	4
**Organization/clarity of presentation**	“The organization of the PowerPoint was really good. It was kind of bite-sized.”	3
	“It was pretty easy to pay attention…the knowledge was easily distributed.”	2

**Table 3.** Social congruence themes.

Theme	Representative Quotes	Student #
**Informal, approachable tone; reduced intimidation**	“It wasn’t intimidating at all.”	2
	“I think it set a more causal tone for the lesson and honestly I think it enhanced my engagement.	4
**Comfort asking questions; psychological safety**	“Definitely felt more comfortable asking them questions compared to faculty.”	9
	“[The session was] more like an even playing field, easier to be wrong in front of them.”	8
	“They know how it feels to be up there…[I] felt more comfortable to be wrong.”	6
**Engagement through interactive, fun elements**	“I would say [I was] more engaged.”	4
	“It just seemed fun to engage because everybody was.”	8
	“They made it more interactive…not just talking at us.”	6
**Relatability and trust from shared role**	“I have a lot of trust in them…I’ve known those people for a year.”	6
	“Peer-to-peer…helped me. It felt more personable….forced me to pay more attention.	3

Other themes included increased engagement, enhanced comfort with material, and several students highlighted the value in practical training by near-peers. As one student explained, ‘Anything that involves us actually doing outside of just talking…would get a great benefit from…peer-to-peer [teaching]’ (student #10). Additionally, another student described, ‘[For] trauma scenarios, emergency skills, our classmates may have had experience as EMS or EMTs that would be beneficial’ (student #6).

## Discussion

Our findings suggest that a brief, skills-focused near-peer workshop on opioid overdose detection and naloxone administration was associated with higher student knowledge scores and more favorable attitudes toward opioid overdoses compared to faculty-led sessions. These results are consistent with broader evidence on peer-assisted learning, which highlights the advantages of instructional alignment with learners’ current cognitive frameworks and social context [8,14]. In particular, Loda et al. identify two key factors for creating an environment conducive to learning, namely, easily identifying where students may be struggling and using language familiar with each stakeholder [9].

In our study, the near-peer educator tailored the session to preclinical students’ level, focusing on relevant information to identify an opioid overdose in the community and appropriately manage it. Near-peer teaching also mitigates the traditional power dynamic between professional educator and student which improves engagement. The feeling of equal footing also promotes comfort amongst peers and yields higher engagement [15–17]. By focusing on individual strengths of student peers and sharing them, we can improve the effectiveness of the group as a whole and in turn strengthen the knowledge and engagement toward foundational and clinical topics.

Of note, the differences in professional backgrounds between the faculty-led and near-peer sessions may explain some of the observed outcomes, particularly the Naloxone Use subscale results. The faculty-led sessions were conducted by a board-certified emergency medicine physician and a pharmacist, whereas the near-peer sessions were led by a medical student with four years of paramedic experience. The higher scores on the Naloxone Use subscale among faculty-led participants (80.4% vs 77.9%, *p* < 0.001) may reflect the pharmacist’s specialized expertise in medication administration, pharmacokinetics, and naloxone dosing protocols. Pharmacists receive extensive training in medication delivery systems, drug interactions, and pharmacological mechanisms that may have been communicated more comprehensively during faculty-led sessions. Conversely, the near-peer instructor's paramedic background emphasized field-based emergency response and practical overdose management skills, which may have contributed to the higher scores observed on the Overdose Risk, Signs of Overdose, and Life-Saving Actions subscales. These differing backgrounds highlight that both faculty and near-peer instructors bring complementary strengths to naloxone training, and the optimal approach may involve collaboration between healthcare professionals with diverse clinical backgrounds. Future studies should consider hybrid models that leverage faculty expertise in pharmacology alongside near-peer facilitation of practical skills training.

### Limitations

Our study has several limitations. First, the students were not required to complete the OOKS or the OOAS, so the results may not reflect the knowledge and attitudes of all students who participated in the training. This also affected response rate, and we acknowledge that response rate bias may exist if students who chose to complete the survey differed systematically from non-responders in their knowledge and attitudes. However, the large sample size and sizeable response rate mitigate some concerns regarding responsiveness. Second, the observational design raises potential concerns about the cohorts. We did not collect pre-survey data for the faculty-led cohort as well as baseline characteristics like MCAT scores from post-survey respondents, limiting our ability to compare differences between cohorts prior to the intervention. We acknowledge that this may contribute to the differences between cohorts. Additionally, our study captured data after the training program and did not evaluate knowledge retention and attitudes longitudinally.

Despite these limitations, the mixed-method design provides complementary quantitative and qualitative insights into student learning experiences. The qualitative findings offer a plausible explanatory framework for the quantitative results, suggesting that perceived alignment in knowledge level and social role may influence learner engagement and attitudes. Future research should incorporate prospective, controlled designs with pre- and post-intervention assessments, evaluate longitudinal retention, and further explore how instructor characteristics and learner factors interact to influence educational outcomes.

## Conclusion

A near-peer–facilitated overdose simulation and naloxone training was associated with an improvement in medical students’ knowledge and attitudes and was well received by participants. Programs seeking scalable approaches to expand overdose response competence can integrate structured near-peer roles within existing curricula. Future work should assess long-term retention, performance in authentic clinical/community settings, and implementation across diverse institutions to optimize training fidelity and impact.

## Data Availability

All data collected is summarized within the paper.
